# New Gene Markers of Angiogenesis and Blood Vessels Development in Porcine Ovarian Granulosa Cells during Short-Term Primary Culture In Vitro

**DOI:** 10.1155/2019/6545210

**Published:** 2019-01-30

**Authors:** Błażej Chermuła, Maciej Brązert, Dariusz Iżycki, Sylwia Ciesiółka, Wiesława Kranc, Piotr Celichowski, Katarzyna Ożegowska, Mariusz J. Nawrocki, Maurycy Jankowski, Michal Jeseta, Paweł Antosik, Dorota Bukowska, Mariusz T. Skowroński, Klaus P. Brussow, Małgorzata Bruska, Leszek Pawelczyk, Maciej Zabel, Michał Nowicki, Bartosz Kempisty

**Affiliations:** ^1^Department of Infertility and Reproductive Endocrinology, Poznan University of Medical Sciences, Poznan, Poland; ^2^Department of Cancer Immunology, Chair of Medical Biotechnology, Poznan University of Medical Sciences, Poland; ^3^Department of Histology and Embryology, Poznan University of Medical Sciences, Poznan, Poland; ^4^Department of Anatomy, Poznan University of Medical Sciences, Poznan, Poland; ^5^Department of Obstetrics and Gynecology, University Hospital and Masaryk University, Brno, Czech Republic; ^6^Veterinary Center, Nicolaus Copernicus University in Torun, Torun, Poland; ^7^Division of Histology and Embryology, Department of Human Morphology and Embryology, Wroclaw Medical University, Wroclaw, Poland; ^8^Division of Anatomy and Histology, University of Zielona Góra, Zielona Góra, Poland

## Abstract

The physiological processes that drive the development of ovarian follicle, as well as the process of oogenesis, are quite well known. Granulosa cells are major players in this occurrence, being the somatic element of the female gamete development. They participate directly in the processes of oogenesis, building the cumulus-oocyte complex surrounding the ovum. In addition to that, they have a further impact on the reproductive processes, being a place of steroid sex hormone synthesis and secretion. It is known that the follicle development creates a major need for angiogenesis and blood vessel development in the ovary. In this study, we use novel molecular approaches to analyze markers of these processes in porcine granulosa cultured primarily* in vitro. *The cells were recovered from mature* sus scrofa* specimen after slaughter. They were then subjected to enzymatic digestion and culture primarily for a short term. The RNA was extracted from cultures in specific time periods (0h, 24h, 48h, 96h, and 144h) and analyzed using expression microarrays. The genes that exhibited fold change bigger than |2|, and adjusted p-value lower than 0.05, were considered differentially expressed. From these, we have chosen the members of “angiogenesis,” “blood vessel development,” “blood vessel morphogenesis,” “cardiovascular system development,” and “vasculature development” for further selection.* CCL2, FGFR2, SFRP2, PDPN, DCN, CAV1, CHI3L1, ITGB3, FN1,* and* LOX* which are upregulated, as well as* CXCL10, NEBL, IHH, TGFBR3, SCUBE1, IGF1, EDNRA, RHOB, PPARD, *and* SLITRK5* genes whose expression is downregulated through the time of culture, were chosen as the potential markers, as their expression varied the most during the time of culture. The fold changes were further validated with RT-qPCR. The genes were described, with special attention to their possible function in GCs during culture. The results broaden the general knowledge about GC's* in vitro* molecular processes and might serve as a point of reference for further* in vivo* and clinical studies.

## 1. Introduction

In the Graafian follicle, apart from the oocyte, distinct populations of somatic cells, called the granulosa cells (GCs), can be distinguished. Their functions include androgen to estrogen conversion, as well as synthesis of progesterone. Additionally, they engage in a bidirectional dialog with the oocyte, ensuring its competence for reproduction [1]. They are the innermost of the cells that surround the cumulus-oocyte complex. Processes in which they participate include the oogenesis, oocyte storage, and its maturation, all accompanied by close mutual interactions between the somatic cells and the gamete [2–4]. The granulosa cells provide the microenvironment necessary for development of the follicle and oocyte maturation [5–7]. They also influence the development of the female gamete in a paracrine and autocrine manner [8].

During the luteal phase of the menstrual cycle, the GCs undergo a transformation resulting in the formation of the corpus luteum [9]. Understanding the molecular mechanisms regulating that process is essential for completely understanding the regulation of oogenesis and ovarian function.

One of the effects of pituitary gonadotropin on the GCs is the upregulation of genes associated with the processes of angiogenesis and blood vessel development in the follicular microenvironment. These processes are essential for follicle and corpus luteum development. So far, there have only been a few reports describing genes responsible for blood vessels development, morphogenesis, and blood circulation in porcine oocytes [10]. Because of structural limitation, including the presence of theca layers, it is very interesting whether GCs have the possibility and genetic basis for the development of blood vessels network in the long period of follicular development.

The hormone-producing granulosa functions due to the formation of a complex capillary network in the ovary, which allows for oxygen, nutrient, and precursor transport. Without these, the synthesis and release of the range of ovarian hormones would not be possible. [11]. Several experimental models have demonstrated that ovarian function is critically dependent on angiogenesis during follicular development, ovulation, and corpus luteum function [12]. The inhibition of the angiogenetic and blood vessel development processes causes the impairment of follicle growth and ovulation disruption, as well as significant defects of corpus luteum function and development. [13].

DNA microarrays are a useful diagnostic method to examine changes in gene expression among granulosa cells and oocytes at various developmental stages [7]. The transcriptome analysis of GCs could be a powerful tool to improve our knowledge about the pathways involved in oocyte development [1]. However, to the best of our knowledge, gene expression profiles and identification of mechanisms, in which GCs form vessel microenvironment, have not yet been reported.

In this study, we performed* in vitro* cultures of porcine granulosa cells. We have investigated the transcriptomic profile changes of genes involved in “Angiogenesis,” “Blood vessel development,” “Blood vessel morphogenesis,” “Cardiovascular system development,” and “Vasculature development” during the short-term primary* in vitro* culture.

## 2. Materials and Methods

### 2.1. Animals

40 crossbred gilts belonging to a local Landrace were used in the study. They had a mean age of 170 days and weight of 98kg. The feeding, breeding and housing conditions of all the animals were identical. Poznan University of Medical Sciences Bioethical Committee approved the study by resolution 32/2012.

### 2.2. Collection of Porcine Ovaries and In Vitro Culture of Granulosa Cells

After slaughter, the reproductive tracts of the animal specimen were transported to the laboratory at 38°C in 0.9% NaCl within 30 min. Ovaries (n=80) were extracted from the tracts and placed in fetal bovine serum (FBS; Sigma-Aldrich Co., St. Louis, MO, USA) supplemented PBS. The follicles were visually measured, with those of the estimated diameter greater than 5mm (n=300) opened through puncture with a 5mm syringe and 20-G needle. This operation was performed in a sterile Petri dish, with the cumulus-oocyte complexes (COCs) and follicular fluid (FF) recovered. The GCs were extracted from the aspired follicular fluid, with the COCs discarded. Follicular fluid from each batch (collected from 20 gilts, 40 ovaries) of the specimen was pooled and treated as one biological sample. GCs were extracted from the fluid through centrifugation at 200 x g for 10 min and removal of remnant supernatant. The pellet was then suspended in the culture medium and, after counting, seeded onto culture dishes.

The number of cells per 1 mL of medium was counted using ADAM Automatic Cell counter (NanoEnTek, Pleasanton, CA, USA). The total volume of GC containing medium was then fractioned accordingly to the count readings and seeded onto 25cm^2^ culture bottles (Nunclon, Thermo-Fisher Scientific, Waltham, MA, USA). Dulbecco's Modified Eagle's Medium (DMEM, Sigma-Aldrich, USA), 2% fetal calf serum (FCS) (PAA, Linz, Austria), 10 mg/ml ascorbic acid (Sigma-Aldrich, USA), 0.05 *μ*M dexamethasone (Sigma-Aldrich, USA), 200 mM L-glutamine (Invitrogen, USA), 10 mg/ml gentamycin (Invitrogen, USA), 10,000 units/ml penicillin, and 10,000 *μ*g/ml streptomycin (Invitrogen, USA) were all of the components of the used culture medium. Cell cultivation was conducted at 38.5°C under 5% CO_2_ aerobic conditions. Cell detachment using 0.05% trypsin-EDTA (Invitrogen, USA) for 3 min and sample collection was performed on days 1, 2, 4, and 6 of the culture. The Z2 counter or cell viability analyzer (Vi-Cell XR 2.03; both Beckman Coulter, USA) was used to count the number of cells in samples.

### 2.3. Microarray Expression Analysis and Statistics

The Affymetrix was conducted as previously described by Trejter et al. [14]. RNA was isolated from each sample using the Chomczyński-Sacchi method [15]. Two rounds of sense cDNA amplification were applied to the resulting RNA samples (Ambion® WT Expression Kit). Biotin labelling and fragmentation were applied to the cDNA, Affymetrix GeneChip® WT Terminal Labeling and Hybridization (Affymetrix). The labelled cDNA fragments (5.5 *μ*g) were hybridized to Affymetrix® Porcine Gene 1.1 ST Array Strip (48°C/20 h). The microarrays were then washed and stained, following the Affymetrix GeneAtlas Fluidics Station technical protocol. Imaging Station of GeneAtlas System was then employed to scan the stained chips. After reading, the scanned data was subjected to preliminary analysis using Affymetrix GeneAtlas™ Operating Software. The software provided criteria that were used to check the quality of gene expression data. Downstream data analysis was conducted using the obtained CEL files. Bioconductor and R programming language were used to obtain all of the analyses and graphs. The CEL files were merged with files that contained their description. The Robust Multiarray Averaging (RMA) algorithm was used for background correction, normalization, and a summary of the results. All of the microarrays were performed on 2 biological replicates, 3 technical repeats each. The repeats were averaged to present the best approximate value for each biological replicate. The mean of these values was used as the level of gene expression.

The lists of differentially up- and downregulated genes were uploaded to the DAVID software (Database for Annotation, Visualization and Integrated Discovery) and subjected to enriched Gene Ontology term extraction. Ontology groups that contained at least 5 genes and expressed p-value<0.05 were selected for further analysis. Particularly, the “angiogenesis”, “blood vessel development,” “blood vessel morphogenesis,” “cardiovascular system development,” and “vasculature development” were selected as Gene Ontology Biological Process Terms (GO BPs) of interest. Hierarchical clusterization procedure was applied to gene expression data which allowed presenting them as heatmaps.

The mutual relations between the gene sets of interest were investigated using the GOplot package [16]. Moreover, the package allowed for calculation of the z-score (the number of upregulated genes minus the number of downregulated genes divided by the square root of the count). Analysis of this score allowed for the comparison of selected GO BP terms.

96 genes, belonging to “angiogenesis,” “blood vessel development,” “blood vessel morphogenesis,” “cardiovascular system development,” and “vasculature development” GO BP terms, were the focus of this manuscript. 10 genes with the most upregulated downregulated expression levels were chosen to be analyzed in detail. STRING10 (Search Tool for the Retrieval of Interacting Genes) software allowed for the analysis of the interactions between proteins coded by the genes of interest and the gene itself. Information on protein/gene interactions, that include experimental data, computational prediction methods, and public text collections are all available in that database. Its engine allowed us to generate a molecular interaction network between the genes of interest. The search was based on cooccurrences of genes/proteins in scientific texts (textmining), coexpression, and experimentally observed interactions.

Finally, REACTOME FIViz to the Cytoscape 3.6.0 software was used to analyze the functional interactions between the genes belonging to the chosen gene ontologies. This app serves in finding pathways and network patterns related to cancer and other disease types. It accesses the Reactome database, reading through the stored pathways, which allows for the analysis of gene set pathway enrichment, visualization of hit pathways with the use of manually laid out pathway diagrams, and investigation of functional relationships between the analyzed genes.

### 2.4. Real-Time Quantitative Polymerase Chain Reaction (RT-qPCR) Analysis

RNA samples extracted from the different periods of primary culture of the analyzed cells were subjected to treatment with DNase I and reverse-transcription (RT) into cDNA. LightCycler real-time PCR detection system (Roche Diagnostics GmbH, Mannheim, Germany) was used to perform the RT-qPCR analysis, with SYBR® Green I used as a detection dye, and target cDNA quantification using the relative method. The internal glyceraldehyde-3-phosphate dehydrogenase (GAPDH) standard was used to standardize the relative levels of PTX3, COX2, HAS2, and TSG6 transcripts in all of the samples. 2 *μ*l of cDNA solution, 18 *μ*l of QuantiTect® SYBR® Green PCR (Master Mix Qiagen GmbH, Hilden, Germany), and primers (Table 1) constituted the full PCR reaction mix. The negative control was achieved through processing of one RNA sample from each preparation without PCR reaction.

PBGD and ACTB housekeeping gene levels were used to calculate the relative levels of the GC specific gene expression. 18S rRNA was used as an additional internal standard, allowing the demonstration of lack of differential regulation of the other housekeeping genes in the culture, as it has been defined as an appropriate gene for this task in quantitative PCR studies. All of the RT-qPCR analyses were performed on 2 biological replicates, 3 technical repeats each. The repeats were averaged to present the best approximate value for each biological replicate. The mean of these values was used as the level of gene expression.

### 2.5. Statistical Analysis

Bioconductor and R programming languages (R version 3.5.1) served to perform the statistical analysis of the results in this study. Statistical significance of the gene of interest expression was determined using the moderated t-statistics from the empirical Bayes method. Benjamini and Hochberg's false discovery rate was employed to correct the p-value for multiple comparisons. P<0.05 was considered to indicate a statistically significant difference. The enriched GO term statistical significance test was performed using DAVID database software (v.6.8). The Benjamini method was used for that task. Each GO term was considered significantly enriched if it contained at least 5 differently expressed genes and showed P<0.05. RT-qPCR result statistical analysis was conducted using MS Excel 2016 Real Statistics Resource Pack add-on.

## 3. Results

The changes in the expression of GC genes were analyzed using the expression microarrays, after 2, 4, and 6 days of culture. Presence of 27558 transcripts has been identified. After selection based on fold change higher than |2| and p-value<0.05, a set of 3380 differentially expressed genes has been identified.

The gene ontology terms were extracted with the use of DAVID (Database for Annotation, Visualization and Integrated Discovery) software. Overall, after subjecting up- and downregulated gene sets to the DAVID search and selecting only those of adjusted p-value<0.05, 344 GO BP terms containing the differentially expressed genes have been identified.

**Figure 1:**
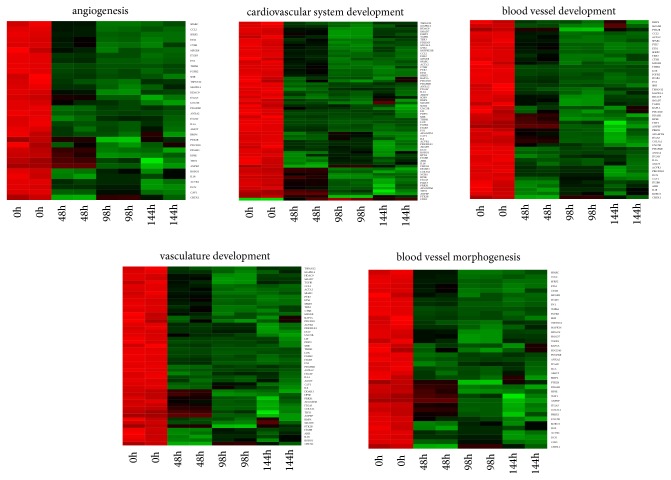
Heat map representation of differentially expressed genes belonging to the chosen “angiogenesis”, “blood vessel development”, “blood vessel morphogenesis”, “cardiovascular system development” and “vasculature development” GO BP terms. Arbitrary signal intensity acquired from microarray analysis is represented by colours (green, higher; red, lower expression). Log2 signal intensity values for any single gene were resized to Row Z-Score scale (from -2, the lowest expression to +2, the highest expression for the single gene). All of the time periods presented twice to show the results in both of the analyzed biological replicates.

“Angiogenesis,” “blood vessel development,” “blood vessel morphogenesis,” “cardiovascular system development,” and “vasculature development” gene ontologies were the focus of this paper. Hierarchical clusterization procedure was applied to these gene sets, which allowed for presenting their changes in expression as heatmaps (Figure 1). Additionally, Table 1 was compiled to provide the complete gene symbols, fold changes in expression, Entrez gene IDs, and corrected p-values of these genes.

**Figure 2:**
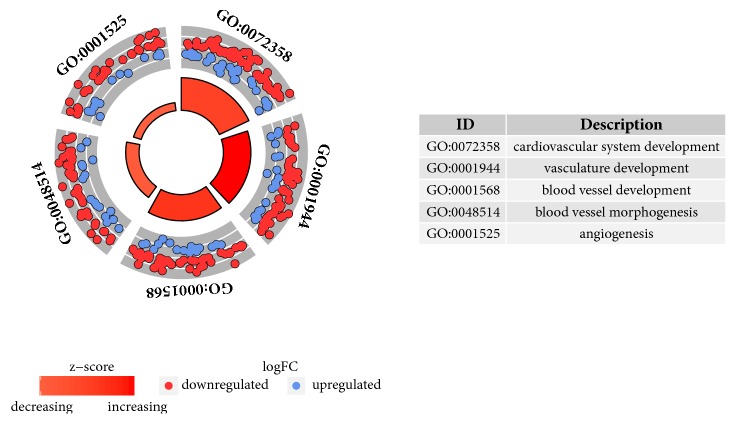
The Circular visualization of the results of gene-annotation enrichment analysis. The outer circle shows a scatter plot for each term of the logFC of the assigned genes. Red circles display up-regulation and blue ones down-regulation. The inner circle is the representation of Z-score. The size and the colour of the bar correspond to the value of z-score.

**Figure 3:**
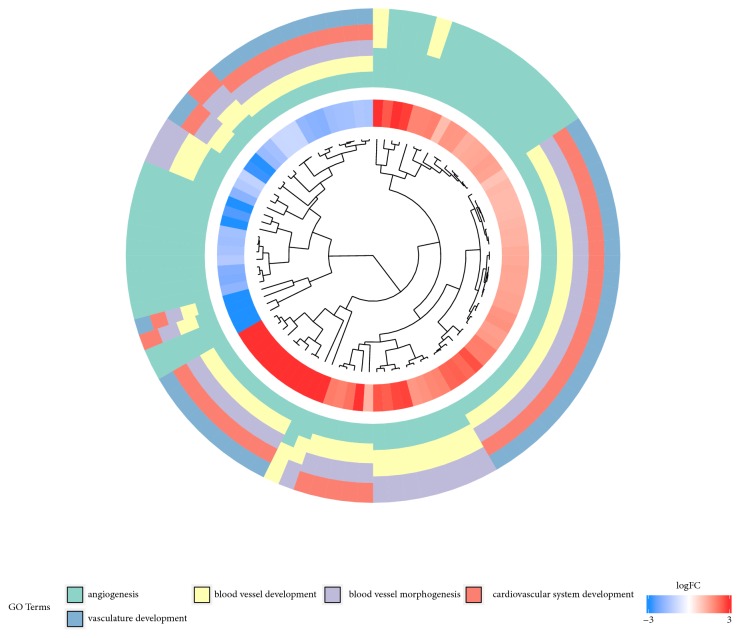
The Representation of hierarchical clusterization, fold change and assignment of differently expressed genes that belong to chosen “angiogenesis”, “blood vessel development”, “blood vessel morphogenesis”, “cardiovascular system development” and “vasculature development” GO BP terms. Genes are grouped together based on their expression patterns and the clusterization pattern is represented by dendrogram inside the circle. The middle ring represents the logarithm of gene expression fold change of studied genes. The outer ring represents the terms assigned to the genes.

The enrichment levels of all of the GO BP terms of interest were further analyzed, being expressed as a z-score and presented in a form of circular visualization (Figure 2). The gene expression profiles were then hierarchically clustered to improve the understanding of the interactions between chosen gene ontologies. The results, in the form of dendrogram combined with fold changes of studied genes, are presented as Figure 3.

From the 96 differentially expressed genes of interest, 10 most upregulated and 10 most downregulated ones were chosen for the downstream analysis.

**Figure 4:**
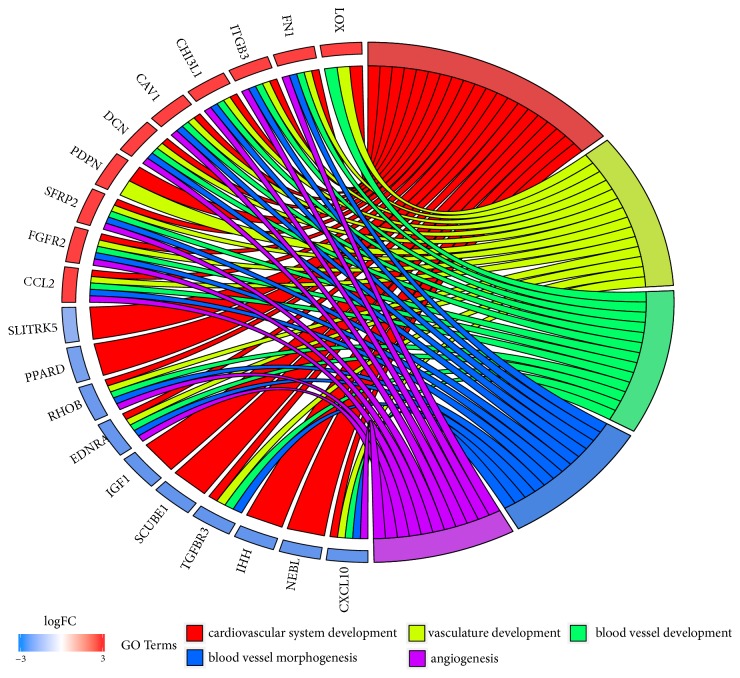
The representation of the mutual relationship between 10 most upregulated and 10 most downregulated genes that belong to “angiogenesis”, “blood vessel development”, “blood vessel morphogenesis”, “cardiovascular system development” and “vasculature development” GO BP terms. The ribbons indicate which gene belongs to which categories. The genes were sorted by logFC from most to least changed gene.

**Figure 5:**
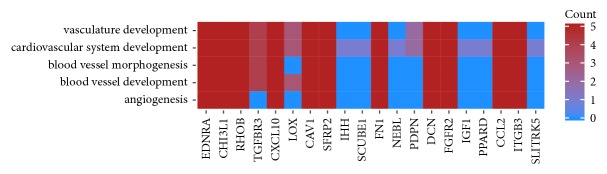
Heatmap showing the gene occurrence between 10 most upregulated and 10 most downregulated genes that belong to “angiogenesis”, “blood vessel development”, “blood vessel morphogenesis”, “cardiovascular system development” and “vasculature development” GO BP terms.

As genes can belong to multiple gene ontologies, the intersections of genes between the selected GO BPs were analyzed. The results of that analysis were presented as a circle plot (Figure 4) and heatmap (Figure 5).

**Figure 6:**
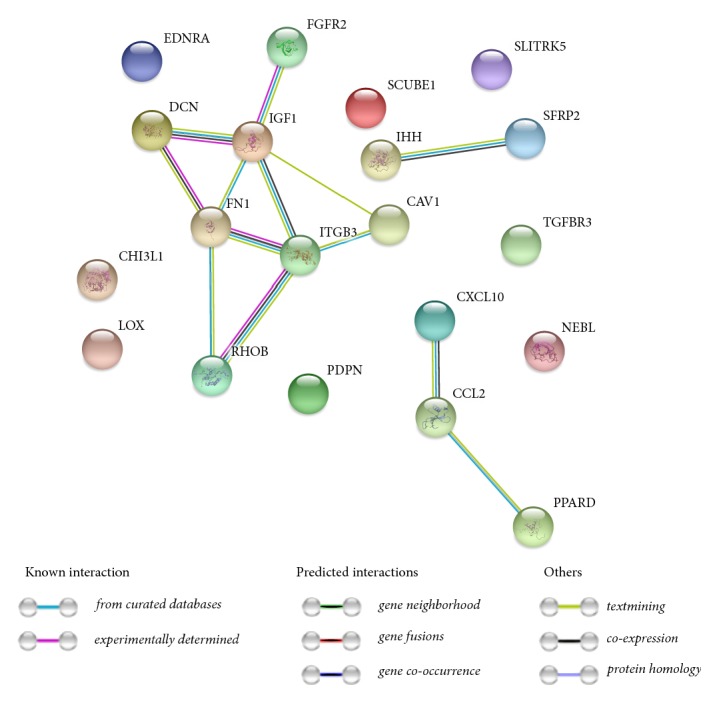
STRING-generated interaction network among differentially expressed genes belonging to the between 10 most upregulated and 10 most downregulated genes that belong to “angiogenesis”, “blood vessel development”, “blood vessel morphogenesis”, “cardiovascular system development” and “vasculature development” GO BP terms. The intensity of the edges reflects the strength of the interaction score.

**Figure 7:**
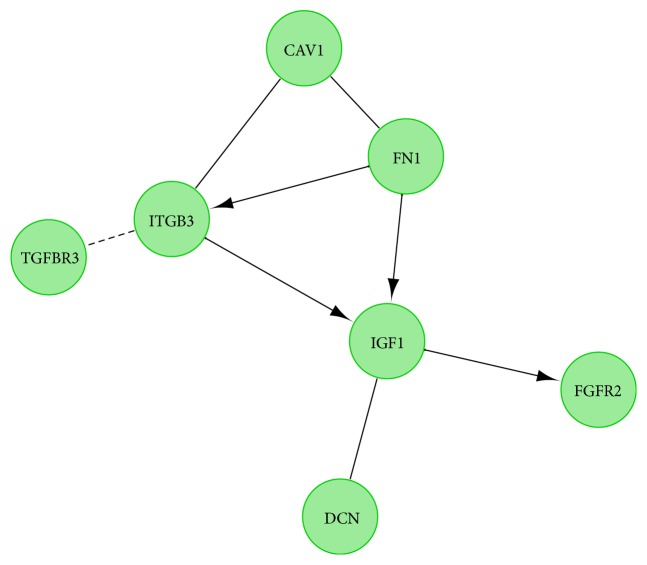
Functional interaction (FI) between 10 most upregulated and 10 most downregulated genes that belong to “angiogenesis”, “blood vessel development”, “blood vessel morphogenesis”, “cardiovascular system development” and “vasculature development” GO BP terms. In the following figure “->” stands for activating/catalyzing, “-|” for inhibition, “-” for FIs extracted from complexes or inputs, and “---” for predicted FIs.

Next, a molecular interaction network between the genes of interest was investigated using STRING software. The results, based on several association criteria, were presented as a STRING graph (Figure 6.). Finally, the functional interactions between the genes of interest were investigated using REACTOME FIViz app to Cytoscape 3.6.0 software. The results were presented as Figure 7.

**Figure 8:**
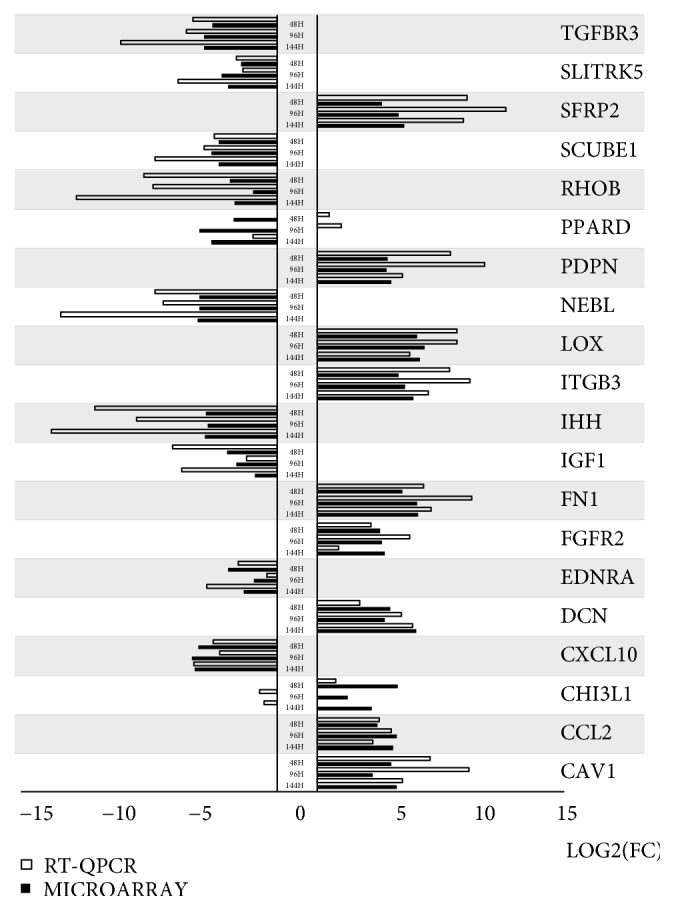
Fold changes of gene expression obtained through RT-qPCR analysis compared to the output of expression microarrays, presented as a bar graph.

Additionally, all of the microarray results were validated using RT-qPCR. The results were compared and presented as a bar graph (Figure 8). As can be seen, in most examples the direction of changes was confirmed. The only exceptions are PPARD and CHI3L1. First of these genes showed the different direction of expression change in hours 48 and 96, and the same direction as microarrays in hour 144. The second gene showed the same direction of changes in expression in hour 48, with the results differing between the two methods in hours 96 and 144. On the other hand, the scale of change in gene expression varied when analyzed with microarrays and RT-qPCR. In some examples, the variation was very small (DCN, CXCL10), while some genes showed a significant difference (IHH, NEBL, and SFRP2).

## 4. Discussion

In the growing ovarian follicle, in addition to the oocyte, separate somatic cell populations are also present. Theca cells, forming internal and external theca layers, reside outwardly and are separated from the internal layer of granulosa cells through the basal membrane. Under the influence of blood flow, from penetrating vessels to the basement membrane of the follicle, both of these layers are gradually differentiated [6]. Theca and granulosa cells participate in and promote the formation of corpus luteum. Theca cells are only correlated with the development of ovarian follicles. Two distinct granulosa cell populations exist in the mature vesicle: mural granulosa cells (GCs) and cumulus cells (CCs) directly related to the oocyte forming cumulus-oocyte complexes (COCs) [17]. GCs play an important role in folliculogenesis and ovarian follicle development process. Along with the development of the gap junction connections, they connect between themselves and with the oocyte. Bidirectional communication with the oocyte ensures the provision of nutrients essential for oocyte development. In addition, the natural roles of GCs include hormonal activity, with the production of estradiol during follicular growth and secretion of progesterone after ovulation [18]. The development of ovaries and ovarian follicles requires the expression of different genes appropriate for each developmental stage.

The development of new approaches, such as microarrays, opened new insights into GC functions and pathways that are activated during follicle development [19]. The microarray technique provides a global analysis of the transcriptome of a tissue and is a hypothesis-generating tool [20].

In our research, we paid particular attention to the study of the granulosa cells' properties and their predisposition to participate in the angiogenesis and blood vessels development process in maturing ovarian follicle. A special focus was put on GCs that turn into ovarian corpus luteum. The growth and development of microvessels are extremely fast in female reproductive tissues. Hence, these tissues are highly vascularized in their mature state. Due to high vascularity, female reproductive tissues, especially corpus luteum, receive one of the highest blood flow rates per unit of tissue. The ovarian corpus luteum plays a key role in reproduction because it is the main source of circulating progesterone. After ovulation, corpus luteum, constructed among others from GCs, grows and vascularizes extremely quickly. In fact, the rate of tissue growth, development, and the process of corpus luteum angiogenesis are even faster than those of growing tumours [21]. The role of angiogenesis is important in solid tumour growth processes, as thanks to the increase of blood supply tumour growth is more intense [22–25]. Thus, analysis of the genetic background and predicting of genes responsible for formation and development of blood vessels in GCs guarantee obtaining an excellent model for testing factors which regulate the process of angiogenesis and formation of new blood vessels. These are a critical moment for proper growth, development, and functioning of this ovarian tissue.

The purpose of this study was to analyze gene expression in porcine GCs, in order to define differentially expressed genes belongs to the “angiogenesis,” “blood vessel development,” “blood vessel morphogenesis,” “cardiovascular system development,” and “vasculature development” ontology groups during the long-term primary* in vitro* culture. This data points to a key role of genes belonging to these functional groups.

During our study, we have selected 10 most upregulated and 10 most downregulated genes that belong to these five ontology groups. In our research, by using microarray approach, we aimed to investigate the transcriptome profile of porcine granulosa cells, using total RNA isolated from before the primary culture and after 48h, 96h, and 144h of in vitro cultivation.

From 96 differently expressed genes belonging to the chosen ontology groups, we have selected* CCL2, FGFR2, SFRP2, PDPN, DCN, CAV1, CHI3L1, ITGB3, FN1, *and* LOX* which are upregulated, as well as* CXCL10, NEBL, IHH, TGFBR3, SCUBE1, IGF1, EDNRA, RHOB, PPARD, *and* SLITRK5* genes whose expression is downregulated through the time of culture.

The most upregulated gene, belonging to the three of five analyzed ontology groups, is* LOX* (*Lysyl oxidase*) gene. We can find this gene in “blood vessel development,” “cardiovascular system development,” and “vasculature development” ontology groups.* LOX* encodes an extracellular amine oxidase, the primary function of which is the posttranslational modification of collagen and elastin in ECM (extracellular matrix). Thereby, it catalyzes covalent fibre cross-linking which may also occur during vessel formation [26]. Other upregulated genes, except for* PDPN* (*Podoplanin*), which represents only the “cardiovascular system development” and “vasculature development” ontology group, belong to all five analyzed groups. A study by Shindo et al. suggested that* PDPN* expression in stromal fibroblasts in pancreatic cancer was reported to be associated with vascular invasion [27]. This finding may indicate the importance of this gene in the blood vessels formation and rapid growth of cellular structures, which are undoubtedly specific for cancerous cells as well as granulosa cells examined by us. The next most upregulated gene just after* LOX* is* FN1* (*Fibronectin 1*). Fibronectin (Fn) is one of the most abundant proteins of the cardiovascular system. It is a part of the fibrous extracellular matrix supporting endothelial cells. It also occurs, in a soluble form, at high concentrations in the blood [28]. Fn fibres form linear and branched meshes around cells and connect to neighbouring cells [29]. This protein exists in many isoforms generated by alternative splicing. For example, EIIIA/EIIIB double-null mice exhibit a requirement for these domains in the development of blood vessels during embryogenesis [28]. FN1 protein activates proteins encoded by* ITGB3* (*Integrin subunit beta 3*), the next upregulated gene. Integrin signaling regulates various angiogenetic functions [30].* ITGB3* shows coexpression with* FN1* gene and two of the analyzed, downregulated genes which are* IGF1* (*Insulin-like growth factor 1*) and* RHOB* (*Ras homolog family member B*).* RHOB* was postulated to act as a tumour suppressor and regulate apoptosis. Overexpression of this gene inhibits proliferation, migration, and invasion of gastric carcinoma cells [31]. Gerald et al. have shown that loss of RHOB reduced the extent of pathological angiogenesis in the ischemic retina and led to reduced angiogenesis in response to skin wounds [32]. Several lines of evidence suggest that normal* IGF-1* expression, contained only in the cardiovascular system, stimulates vascular health later in life [33]. This may explain down-expression of this gene in* in vitro* cultivated GCs. The IGF1 protein interacts with proteins encoded by two upregulated genes:* DCN* (*Decorin*) and* FGFR2* (*Fibroblast growth factor receptor 2*). It is believed that DCN acts with IGF1, creating a protein complex, and it is involved in the regulation of angiogenesis with contradictory results. In terms of cancer, DCN has been shown to have a negative impact on tumour angiogenesis [34]. On the other hand, DCN has been shown to play a proangiogenic role, allowing adhesion of endothelial cells to type I collagen and *α*1*β*2 integrin, supporting the interaction between integrin and collagen [35]. Thus, considering our results and previous reports, it can be presumed that the effect of proangiogenic and antiangiogenic DCN activity depends mainly on the cellular and molecular microenvironment in which angiogenesis occurs. FGFR2 is a protein activated by the IGF1 protein. House et al. demonstrated that FGFR2 acts in endothelial cells, promoting cardiac functional recovery and vascular remodelling [36].* CAV1* (*Caveolin 1*) functions have been thoroughly examined using mouse knockouts. Work in this animal model has underlined the importance of CAV1 in modulating proliferation and vascular homeostasis [37]. Caveolin 1 may coexist with FN1 and ITGB3 proteins, forming two interactive complexes. CAV1 has also been shown to regulate the activity of key modulators of vasoactive pathways in endothelial cells. CAV1 deficiency affects the growth of structural disorders of blood vessels and induces pulmonary hypertension in mice and humans [38].* CHI3L1* (*Chitinase 3 like 1), *also named* YKL-40,* is a secreted glycoprotein. It acts as an angiogenic factor to promote tumour angiogenesis in GBM (malignant glioblastoma) and breast cancer. YKL-40 is an important factor that strongly induces angiogenesis of endothelial cells. Combined antiangiogenic therapies targeted against* CHI3L1* and* VEGF* can significantly reduce tumour angiogenesis and consequently inhibit tumour growth [39]. The last two upregulated genes are* SFRP2* (*Secreted frizzled-related protein 2*) and* CCL2* (*C-C motif chemokine ligand 2*). Both of them show a similar level of expression during GCs' long-term* in vitro* culture. Previous studies of* SFRP2* only indicate that this gene is overexpressed in the vascular system of 85% of patients with breast cancer. Hence, its expression is closely related to cancer development [40]. Interestingly* SFRP2* and* CCL2* show coexpression with genes belonging to the downregulated group.* CCL2* interacts with* CXCL10* (*C-X-C motif chemokine ligand 10*) and* PPARD* (*Peroxisome proliferator-activated receptor delta gene*), while* SFRP2* acts with* IHH* (*Indian hedgehog*).* IHH* and* PPARD* belong to “cardiovascular system development” ontology group. Interestingly, it has been shown that progesterone rapidly induces* IHH* expression in the mouse endometrium [41, 42]. This may explain the decrease in expression of this gene during long-term* in vitro* culture carried out without the addition of this hormone.* SCUBE1* (*Signal peptide CUB domain and EGF like domain containing 1*),* NEBL* (*Nebulette*), and* SLITRK5* (*SLIT and NTRK like family member 5*) belong to one and the same ontological group, similarly, to* IGF1*,* PPARD*, and* IHH*.* SCUBE1* was first identified in vascular endothelial cells and is released, in the presence of activated platelets, in the circulatory system [43].* NEBL* encodes a nebulin-like protein that is abundantly expressed in the myocardium. This protein may be involved in cardiac myofibril assembly [44].* SLITRK5* is expressed primarily in nervous tissues, exhibiting neurite-modulating activity [45].* TGFBR3* (*Transforming growth factor beta receptor 3*) plays an important role in epicardial development and coronary artery growth [46]. Decreased expression of this receptor has been observed in various cancers. The last downregulated gene, from those analyzed, is* EDNRA* (*Endothelin receptor type A*). This gene is expressed in all five groups, showing no correlation with the previously tested genes.* EDNRA* controls the production of the type A endothelin receptor and is expressed primarily in vascular muscle cells, regulating their vasoconstriction. Animal models have shown that the expression of the EDNRA protein is significantly reduced in the uteroplacental vascular bed [47]. Most of the downregulated genes are specific for cardiovascular system development and other tissues. This finding explains their expression reduction in GCs' primary* in vitro* culture.

In conclusion, we identified several genes in porcine ovarian granulosa cells that were differentially expressed during the short-term primary* in vitro* culture. The presented data show, for the first time, variable levels of these genes' expression and morphological changes in short-term* in vitro* culture of those cells. We have extracted two group of genes belonging to five ontological groups: “angiogenesis” (GO:0001525), “blood vessel development” (GO:0001568), “blood vessel morphogenesis” (GO:0048514), “cardiovascular system development” (GO:0072358), and “vasculature development” (GO:0001944) containing genes that are primarily responsible for the formation of new blood vessels. One of these groups consists of genes upregulated during cultivation, which may be the markers of these processes in the cultured GCs. As we mentioned above, these genes are mostly specific for forming cancerous tumours. Our research is in line with previous reports about similar dynamics and developmental potential of GCs and cancer cells. This is manifested by the activation of genes that directly take part in the formation of new blood vessels. It may reflect the ability of GCs to rapidly proliferate and differentiate into corpus luteum. These results will help in further elucidating the molecular basis and functional meaning of several gene markers involved in angiogenesis and blood vessel development in ovarian tissue.

## Figures and Tables

**Table 1:** Gene symbols, fold changes in expression, Entrez gene IDs and corrected p values of studied genes.

Official gene symbol	Fold Change 0/48h	Fold Change 0/99h	Fold Change 0/144h	adj.p.Value 0/48h	adj.p.Value 0/98h	adj.p.Value 0/144h	ENTREZ GENE ID
CXCL10	0,036688989	0,027743701	0,030978824	3,47E-06	1,07E-06	1,53E-06	3627
NEBL	0,037427727	0,037303149	0,034729316	9,56E-07	3,68E-07	4,09E-07	10529
IHH	0,049770928	0,053799916	0,047737803	2,16E-06	1,06E-06	1,09E-06	3549
TGFBR3	0,065878161	0,045287893	0,046219886	4,56E-07	1,51E-07	1,50E-07	7049
SCUBE1	0,084451185	0,063451318	0,087117768	4,12E-06	1,09E-06	2,53E-06	80274
IGF1	0,119840598	0,180780671	0,391369389	9,96E-05	0,000172259	0,006283505	3479
EDNRA	0,127283197	0,382041441	0,243107411	2,22E-06	4,49E-05	7,90E-06	1909
RHOB	0,136537436	0,360612176	0,163008697	4,89E-06	7,24E-05	4,71E-06	388
PPARD	0,156457705	0,037874236	0,06167045	8,24E-06	3,18E-07	7,27E-07	5467
SLITRK5	0,219126224	0,096198604	0,124646996	1,47E-05	9,47E-07	1,93E-06	26050
CCL2	12,31754532	28,04020648	24,4792584	1,00E-06	1,62E-07	2,27E-07	6347
FGFR2	14,02831687	15,21343049	17,30951427	1,48E-06	5,90E-07	5,70E-07	2263
SFRP2	15,34470714	30,79201834	38,31992631	9,81E-07	1,80E-07	1,50E-07	6423
PDPN	18,9888844	18,65575256	22,39775328	2,14E-05	1,11E-05	1,07E-05	10630
DCN	21,58631608	16,85223436	63,38248464	4,36E-07	2,52E-07	9,57E-08	1634
CAV1	22,3155745	10,43320303	28,12125816	4,28E-07	4,59E-07	1,50E-07	857
CHI3L1	29,63908063	3,553560954	10,03557976	1,36E-05	0,001433668	7,23E-05	1116
ITGB3	29,91254593	39,85198954	57,68211801	3,52E-07	1,21E-07	9,57E-08	3690
FN1	35,37896196	66,16510016	68,74184776	3,59E-07	9,92E-08	9,57E-08	2335
LOX	67,33838239	88,95072609	75,58491503	2,15E-07	9,14E-08	9,57E-08	4015

## Data Availability

The data used to support the findings of this study are available from the corresponding author upon request.
